# Movement Disorders Associated with COVID-19

**DOI:** 10.1155/2021/3227753

**Published:** 2021-11-08

**Authors:** Mehri Salari, Bahareh Zaker Harofteh, Masoud Etemadifar, Nahad Sedaghat, Hosein Nouri

**Affiliations:** ^1^Department of Neurology, Shahid Beheshti University of Medical Sciences, Tehran, Iran; ^2^Alzahra Research Institute, Alzahra University Hospital, Isfahan University of Medical Sciences, Isfahan, Iran; ^3^Network of Immunity in Infection Malignancy and Autoimmunity (NIIMA), Universal Scientific Education and Research Network (USERN), Isfahan, Iran

## Abstract

As neurological complications associated with COVID-19 keep unfolding, the number of cases with COVID-19-associated de novo movement disorders is rising. Although no clear pathomechanistic explanation is provided yet, the growing number of these cases is somewhat alarming. This review gathers information from 64 reports of de novo movement disorders developing after/during infection with SARS-CoV-2. Three new cases with myoclonus occurring shortly after a COVID-19 infection are also presented. Treatment resulted in partial to complete recovery in all three cases. Although the overall percentage of COVID-19 patients who develop movement disorders is marginal, explanations on a probable causal link have been suggested by numerous reports; most commonly involving immune-mediated and postinfectious and less frequently hypoxic-associated and ischemic-related pathways. The current body of evidence points myoclonus and ataxia out as the most frequent movement disorders occurring in COVID-19 patients. Some cases of tremor, chorea, and hypokinetic-rigid syndrome have also been observed in association with COVID-19. In particular, parkinsonism may be of dual concern in the setting of COVID-19; some have linked viral infections with Parkinson's disease (PD) based on results from cerebrospinal fluid analyses, and PD is speculated to impact the outcome of COVID-19 in patients negatively. In conclusion, the present paper reviewed the demographic, clinical, and treatment-associated information on de novo movement disorders in COVID-19 patients in detail; it also underlined the higher incidence of myoclonus and ataxia associated with COVID-19 than other movement disorders.

## 1. Introduction

Coronavirus disease 2019 (COVID-19), a lower respiratory infection with severe acute respiratory syndrome coronavirus 2 (SARS-CoV-2), is associated with a wide range of neurological manifestations. Most frequent neurological symptoms are nonspecific and include headache, dizziness, disturbance of smell and taste, and myalgia [1, 2]. Among the less common yet more severe complications are cerebrovascular diseases, encephalitis, encephalopathy, and inflammatory central and peripheral nervous systems (CNS and PNS) disorders [3]. Movement abnormalities such as ataxia and opsoclonus-myoclonus have also been observed in COVID-19 patients [4, 5].

Hypothetically, there are multiple entry routes for the virus into the CNS (e.g., trans-synaptic, hematogenous, and lymphatic pathways) [6]. However, the expression of angiotensin-converting enzyme 2 (ACE2) in the human brain parenchyma and whether SARS-CoV-2 infects the CNS neuronal cells is not established yet [7]. Although low to very-low concentrations of viral RNA have been detected by reverse transcription-polymerase chain reaction (RT-PCR) in some autopsied brain samples—especially those from the olfactory bulb and medulla; lack of correlation with microglial activation and nodules in those specimens is against the speculated CNS tropism [8, 9]. Nevertheless, direct neuronal invasion is not the only way SARS-CoV-2 may contribute to neurodegeneration and neuroinflammation. Meanwhile, several observations of movement abnormalities development or worsening in COVID-19 patients have been reported (reviewed later in this paper), which sounds alarming. We review all published reports on COVID-19-associated movement disorders and present three new patients with myoclonus after contracting COVID-19.

## 2. Literature Search Strategy and Inclusion Criteria

We conducted a comprehensive systematic literature search in PubMed and Scopus databases using the following terms: “coronavirus disease 2019,” “COVID,” “COVID-19,” “SARS-CoV-2,” “Neurologic,” “Movement disorders,” “Tremor,” “Myoclonus,” “Parkinsonism,” “ataxia,” “dystonia,” and “chorea”. The Mendeley application was used to detect, scan, and remove duplicates. The remaining studies were scanned for relevant titles/abstracts. All relevant articles that reported patients who developed a movement disorder during/after a SARS-CoV-2 infection were included. Reference lists of the obtained articles were searched for additional relevant results. Any movement disorders in COVID-19 patients were reviewed; demographic information and data on clinical characteristics, treatments applied, and the outcome of patients were extracted. We included case reports, case series, and observational studies published in peer-reviewed journals and preprints available in English. Articles without full texts and studies without laboratory confirmation (RT-PCR) of COVID-19 diagnoses were excluded.

## 3. Results

Our extensive search in the literature yielded a final number of 43 publications reporting movement disorders in 64 patients with SARS-CoV-2 infection (Table 1). Demographic information and data on their medical history, COVID-19 infection severity, movement disorders, treatment, and outcome are presented in Table 1. Furthermore, we added 3 cases of movement disorders in COVID-19 patients from our center to the literature, reported below.

### 3.1. Case 1

A previously healthy 42-year-old man presented with difficulty speaking, cognitive deficits, ataxia, and progressive disabling jerks in upper and lower limbs, leading to falls (see Supplementary video-1). Neurological examination demonstrated spontaneous, action-induced, and tactile-sensitive myoclonus on the face, upper extremities, and lower extremities. He had dysmetria and dysdiadochokinesia with superimposed action-induced myoclonus in the upper and lower extremities. Myoclonus prevented him from standing independently, and he had a wide-based gait. The rest of the neurological examination was normal. Laboratory tests including renal function, liver function, ammonium, urea, and CO2 were normal, except for the nasopharyngeal RT-PCR test for SARS-CoV-2, which was positive. Computed tomography (CT) of the head and brain magnetic resonance imaging (MRI) were normal. Electroencephalogram (EEG) and electromyography (EMG) were performed but did not reveal any abnormalities. Given these findings and the positive SARS-CoV-2 PCR, myoclonus was considered postinfectious. No other symptoms such as fever, myalgia, cough, fatigue, hyposmia, or hypogeusia were present. Cerebrospinal fluid (CSF) analysis was unremarkable, RT-PCR test for SARS-CoV-2 and autoantibodies were negative. He was treated with methylprednisolone 1000 mg IV daily for five days, bringing about a partial resolution of myoclonus. Complete recovery was observed on a follow-up session one month later.

### 3.2. Case 2

A previously healthy 52-year-old man presented with progressive imbalance and generalized jerks (see Supplementary video-2). A week before, he had experienced myalgia and tested positive for SARS-CoV-2. Upon admission, he had generalized stimulus-sensitive myoclonus affecting his face, hands, and legs, confining him to a wheelchair. Other neurological findings, including eye movements, muscle tone, and deep tendon reflexes, were normal. He did not have any coughing, fever, dyspnea, and O2 saturation was more than 97% without nasal O2. Ground-glass opacities suggestive of SARS-CoV-2 infection were detected on chest CT. Laboratory studies showed no increase in inflammatory markers in peripheral blood; CRP and white blood cell count were normal. Brain CT and MRI were normal. Results from CSF evaluations, including autoantibody investigations, were unremarkable. The cerebellar syndrome improved after high-dose IV methylprednisolone (1 gr/day for five days); on a follow-up session after three weeks, the patient could walk independently.

### 3.3. Case 3

A 38-year-old man was referred to our clinic with generalized jerks in his hands (see Supplementary video-3). A week before, he had been discharged from the hospital after four days of hospitalization due to a positive nasopharyngeal RT-PCR test and evident involvement of his lungs. Four days after discharge, he developed fine jerks in his hands and could not write; three days later, he was referred to our clinic with progression and generalization of jerks. He had generalized myoclonus in upper and lower limbs and bizarre abnormal movements that diminished with distraction; he was also restless and seemed anxious. The rest of the neurological examinations were normal. Lab tests were unremarkable. Laboratory investigations and brain MRI scans were normal. CSF analyses, including cell count, protein, and autoimmune panel, were normal. Clonazepam (2 mg/day), levetiracetam (1000 mg/day), and IV methylprednisolone for 5 days (1 g/day) were started, resulting in partial improvement. After two weeks, myoclonus was resolved; functional movements improved with some residual deficits, but he was still suffering from anxiety.

## 4. Discussion

Our understanding of COVID-19-associated neurological manifestations is evolving. With about 200 million confirmed cases of COVID-19, a relatively limited, yet concerning, number of cases of post-/parainfectious movement disorders—most common of which were myoclonus and cerebellar ataxia—have been reported. The overall frequency of abnormal movements in hospitalized COVID-19 patients may not exceed one percent; it was reported as 0.7% (6/841) [51]. All six had hyperkinetic movements (mostly myoclonic tremor; the mean interval between COVID-19 onset and abnormal movements, 3–8 days). Further, we identified numerous cases of movement abnormalities occurring during or after a COVID-19 infection in the literature (reviewed in Table 1). In most cases, the underlying pathomechanisms were assumed to involve immune-mediated and postinfectious processes by the reporting authors. On the other hand, few cases were reportedly caused by hypoxia (case no. 27 [20]) or ischemic strokes (cases no. 46 [35] and 65 [50]); thus, in some instances, patients required different therapeutic approaches. We added three more cases of myoclonus development after COVID-19 infection, furthering our understanding of the neurological manifestations associated with SARS-CoV-2 infection.

### 4.1. Myoclonus and COVID-19

Infection-associated myoclonus can often be encountered among patients with severe viral, bacterial, or parasitic infections and is characterized by abrupt, brief, and sometimes repetitive contractions of the trunk, limbs, or face muscles [16, 25]. Myoclonus is reportedly the most common movement disorder in hospitalized COVID-19 patients [52]. Given the numerous cases of myoclonus in patients without a remarkable history of abnormal movements (see Table 1) and the close temporal association of myoclonus with COVID-19 symptoms, one could suspect a causal link between the two conditions. Some have also advised monitoring COVID-19 survivors for probable, unfortunate posthypoxic myoclonus as a long-term COVID-19 complication [20]. Mechanistically, SARS-CoV-2 infection is speculated to cause myoclonus through one or any combination of the following: (I) hypoxia and systemic (e.g., metabolic) disturbances and (II) direct invasion of the CNS cells and (III) immune-mediated pathways, such as molecular mimicry with the host's CNS tissue [24].

### 4.2. Tremor and COVID-19

Tremor is another type of COVID-19-associated abnormal movement observed in hospitalized COVID-19 patients [52]. We identified 9 cases of tremor occurring in COVID-19 patients, whose clinical and treatment-associated data are described in Table 1 (cases no. 5–10, 25, 39, and 49) [11, 12, 18, 29, 37]. Tremors in PD patients may also exacerbate as a result of a COVID-19 infection [53].

### 4.3. Ataxia and COVID-19

Despite having been rarely attributed to infections before this pandemic, numerous cases of cerebellar ataxia in COVID-19 patients (during or after their infection course) have raised concerns regarding a potential causal link probably through immune-mediated pathomechanisms [25, 54].

### 4.4. Chorea Associated with COVID-19

The most recognized chorea associated with infections is Sydenham's chorea, characterized by autoimmunity against the basal ganglia after a streptococcal infection [55]. We identified only one case of chorea in a COVID-19 patient [10], a 14-year-old girl with previously diagnosed Sydenham chorea when she was 11. She reported no disease activity during the past two years, yet she developed chorea on the third day after COVID-19 symptoms onset. The authors could not conclude whether this was a recurrence of her previously silent disease or an immune-mediated newly developed chorea with a causal link to COVID-19 [10]. However, we assume the latter was less likely, with the very short interval between COVID-19 onset and chorea manifestation (3 days) arguing against an autoimmune scenario.

### 4.5. Hypokinesia and Parkinsonism: Considerations in COVID-19

The acute hypokinetic-rigid syndrome has been reported in two cases (cases no. 5 [11] and 13 [15]); both developed a severe course of infection and required intensive care and mechanical ventilation.

Parkinson's disease (PD) is a neurodegenerative disease caused by the destruction of dopaminergic neurons in substantia nigra pars compacta (SNpc). Evidence of viral infections associated with PD exists; thus, vigilant monitoring and investigation of probable links between SARS-CoV-2 infection and parkinsonism may be necessary [7, 56]. On this matter, the presence of antibodies targeting coronaviruses in cerebrospinal fluid of PD patients was more frequently observed when compared to individuals without PD. Further, the proposed ability of coronaviruses in breaching into the CNS via nasal cavity and nasal neuroepithelium can give us more reasons to be concerned [57, 58]. Interestingly, it has been speculated that PD-associated *α*-synuclein retrograde spreading and aggregation from the olfactory bulb to the midbrain and deeper CNS structures—leading to cellular death of the affected tissue—could offer some protection against SARS-CoV-2 breaching into the CNS via the olfactory route [59]. However, the aforementioned neuropathological data from autopsy studies call this hypothesis into question [8, 9]. Regardless of such hypothesized protective effects, PD patients are nevertheless more vulnerable to complications following respiratory infections, and odds for their hospitalization or development of other comorbidities are higher than the general population [60]. Furthermore, parkinsonian symptoms after viral respiratory infections may hint us toward the less-known sides of the disease etiology and pathogenesis [61].

A community-based case-control study on 12 PD patients who contracted COVID-19 documented a substantial risk of motor and nonmotor symptoms worsening associated with mild to moderate degrees of COVID-19, regardless of the patients' age and disease duration [7].

Lo Monaco and colleagues reported a patient with an 8-year history of PD who developed severe generalized dystonia shortly after contracting COVID-19. Improvement was achieved after an increase in her daily dose of levodopa and clozapine [62].

Inversely, PD and its associated disabilities may negatively impact the outcome of COVID-19 illness, along with other known risk factors such as age and hypertension. Indeed, Fasano et al. reported a higher COVID-19 mortality rate in community-dwelling PD patients (i.e., not living in nursing homes) than the general population [63], and Salari et al. showed PD patients have a higher proportion of COVID-19 mortality in comparison with other patients hospitalized for COVID-19 [64].

Some limitations apply to the present review article, such as the keywords selection; some studies that have not used the keywords incorporated in our search strategy, e.g., those that only mentioned “neurological manifestations” without further specification, might have been missed. The literature search has not thoroughly adhered to the standards of a systematic review, albeit being as exhaustive and comprehensive as possible. Furthermore, cross-reference checking for some reviewed articles may have been incomplete. Review papers bear the limitation of their reviewed articles; as discussed earlier, myoclonus may stem from metabolic comorbidities in some cases. Thus, the attribution of myoclonus to COVID-19 by some case studies is a potential limitation of such reports applying to the present review as well. Another limitation was that electrophysiological data were not obtained from two of our cases (i.e., Cases 2 and 3).

## 5. Conclusion

This review highlighted the higher incidence of myoclonus and ataxia after COVID-19 infection. Other movement disorders were not common after COVID-19, implying that SARS-CoV-2 mainly affects the brain through immune-mediated pathways rather than a direct invasion of the CNS. Nonetheless, the COVID-19 pandemic can affect the outcome of patients with established movement disorders.

## Figures and Tables

**Table 1:** Summary of demographic information and movement disorders associated with COVID-19.

Case no.	Author, year	Age/sex	Comorbidities or past Hx/COVID-19 respiratory symptoms severity	Abnormal movements (side of predominance if reported) (days after COVID-19 onset or recovery)	MD-associated treatments/outcome
1 [5]	Mao et al., 2020	N/A	NR	Ataxia (NR)	NR

2 [5]	Mao et al., 2020	N/A	NR	Ataxia (NR)	NR

3 [10]	Yüksel et al., 2021	14/F	11 y/o: Sydenham chorea—improved, no activity during past 2 years/nonsevere	Sydenham chorea (3 d.a.o)	Carbamazepine/improvement in 7 days

4 [11]	Méndez-Guerrero et al., 2020	58/M	HTN, DLP/severe: Rq. ICU & MV	Generalized myoclonus, opsoclonus, asymmetric hypokinetic-rigid syndrome w/ocular abnormalities (33 d.a.o)	-/significant improvement in tremor, rigidity, and bradykinesia

5 [12]	Cunha et al., 2020	51/M	Disc herniation/severe: Rq. ICU & MV	Action tremor (POR) (38 d.a.o)	NR

6 [12]	Cunha et al., 2020	67/M	HTN, poliomyelitis/severe: Rq. ICU & MV	Postural and action tremor of upper and lower limbs, orthostatic tremor, cortical and subcortical myoclonus (62 d.a.o)	NR

7 [12]	Cunha et al., 2020	34/M	Hepatitis B (healed), typhoid/severe: Rq. ICU & MV	Postural and action tremor of upper and lower limbs (44 d.a.o)	NR

8 [12]	Cunha et al., 2020)	66/F	HTN, ESRD/severe: Rq. ICU & MV	Jerky tremor of the upper limbs, cortical and subcortical myoclonus (59 d.a.o)	NR

9 [12]	Cunha et al., 2020	48/M	HTN, obesity/severe: Rq. ICU & MV	Postural and action tremor of upper limb (65)	NR

10 [13]	Shah et al., 2021	Middle-aged/M	NR/NR	Opsoclonus, cortical myoclonus, and symmetric cerebellar ataxia of speech, limbs, trunk, and gait (21 d.a.r)	IV methylprednisolone, sodium valproate, clonazepam, and levetiracetam/recovered in a week

11 [14]	Wright et al., 2020	79/M	Asbestosis, nondisabling stroke, mild cognitive impairment, T2DM, HTN, and prostatic hypertrophy/mild	Encephalopathy, gait ataxia (8 d.a.o), opsoclonus w/o myoclonus (13 d.a.o)	-/opsoclonus resolved, encephalopathy progressed, and the patient eventually died (43 d.a.o)

12 [15]	Roy et al., 2021	60/M	HTN, DM, HCL/severe: Rq. ICU & MV	Encephalopathy, hypokinetic-rigid state (soon after infection symptoms onset)	Modafinil, carbidopa/levodopa/continuous improvement

13 [16]	Anand et al., 2020	47/M	HTN, DM, morbid obesity, obstructive sleep apnea/severe: Rq. ICU & MV	Generalized, stimulus-induced myoclonus (4 d.a.o)	Ketamine, dexmedetomidine/myoclonus resolved, but the patient ultimately died due to COVID-19 complications

14 [16]	Anand et al., 2020	28/M	DM, HLP/severe: Rq. ICU & MV	Generalized, stimulus-induced myoclonus (3 d.a.o)	Lorazepam, midazolam, dexmedetomidine/resolved

15 [16]	Anand et al., 2020	73/M	HTN, DM, CKD (stage 3)/severe: Rq. ICU & MV	Torso and upper extremities, stimulus-induced myoclonus (9 d.a.o)	Levetiracetam/resolved

16 [16]	Anand et al., 2020	64/M	HTN/severe: Rq. ICU & MV	Upper extremities, stimulus-induced myoclonus (9 d.a.o)	Dexmedetomidine/resolved
17 [16]	Anand et al., 2020	66/M	HTN, DM, obesity/severe: Rq. ICU & MV	Upper extremities and face, spontaneous myoclonus (2 d.a.o)	Dexmedetomidine/resolved

18 [16]	Anand et al., 2020	72/M	DM/severe: Rq. ICU & MV	Generalized, multifocal, stimulus-induced myoclonus (7 d.a.o)	Valproic acid, levetiracetam, lorazepam, dexmedetomidine/continued, but improved myoclonus
19 [16]	Anand et al., 2020	62/M	HTN/severe: Rq. ICU & MV	Generalized, stimulus-induced myoclonus (7 d.a.o)	Valproic acid, primidone, clonazepam, lorazepam/resolved

20 [16]	Anand et al., 2020	71/M	HTN, CKD (stage 2)/nonsevere	Generalized, action-induced; lingual myoclonus, gait disturbance (7 d.a.o)	Levetiracetam, valproic acid/resolved

21 [17]	Rábano-Suárez et al., 2020	63/M	Generalized anxiety disorder/nonsevere, but required ICU admission due to a myoclonic storm	Asynchronous generalized myoclonus (face and limbs), worsening with action and auditory/tactile stimuli (9 d.a.o)	Levetiracetam, valproic acid, clonazepam/scarce improvement
					Methylprednisolone/slight improvement
					PLEX after myoclonus worsened again/improvement

22 [17]	Rábano-Suárez et al., 2020	88/F	HTN, hypothyroidism, nonfunctioning pituitary adenoma, mild cognitive decline, no Hx of MDs	Generalized myoclonus, similar to case no. 22, but milder (∼21 d.a.o)	Methylprednisolone/resolved

23 [17]	Rábano-Suárez et al., 2020	76/M	-/nonsevere	Generalized myoclonus, similar to case no. 22, but milder (11 d.a.o)	Levetiracetam, clonazepam/no benefit- spontaneous progressive improvement after two weeks

24 [18]	Piscitelli et al., 2020	39/F	-/nonsevere	Lower limb tremor, abnormal movements at rest and while sitting or walking (functional) (7 d.a.o)	Benzodiazepines/no significant improvement, spontaneous gradual resolution later on

25 [19]	Hewan et al., 2021	57/F	Morbid obesity, obstructive sleep apnea, chronic obstructive pulmonary disease, heart failure, and schizoaffective disorder (for which she was taking valproic acid)/severe: Rq. ICU & MV	Mental status deterioration, myoclonus in all extremities and upper torso that occurred spontaneously at rest and would intensify upon passive movements (∼26 d.a.o)	Levetiracetam, clonazepam/no significant improvement
					Ketamine was added/myoclonus improved but she was heavily sedated/ myoclonus returned after ketamine was halted
					Methylprednisolone/recovery

26 [20]	Ros-Castelló et al., 2020	72/F	HTN, asthma/severe: Rq. ICU and oxygen therapy	Progressively disabling myoclonus in upper limbs and negative myoclonus in lower limbs (leading to falls; ∼30 d.a.o)	Low-dose clonazepam/resolved

27 [21]	Faber et al., 2020	35/F	Unremarkable/mild	Akinetic-rigid parkinsonism (10 d.a.o) (Parkinson's disease rating scale part III score: 49)	Levodopa/benserazide/significant improvement (Parkinson's disease rating scale part III score: 32)

28 [22]	Werner et al., 2021	62/M	Unremarkable/nonsevere	Cerebellar syndrome with slightly scanning speech and limb, truncal, and gait ataxia (16 d.a.o) (SARA ataxia score: 14)	High-dose methylprednisolone/gradual improvement (SARA 6 days after treatment: 5, and SARA 4 months after treatment: 1)

29 [23]	Urrea-Mendoza et al., 2021	32/M	-/nonsevere	Opsoclonus, myoclonus, ataxia (12 d.a.o)	Clonazepam, divalproex, oral methylprednisolone/substantial improvement
30 [24]	El Otmani et al., 2021	59/M	Recent under control DM/asymptomatic	Generalized myoclonus in torso, limbs, face, tongue, and larynx, intensified by movement and acoustic stimuli (not clear, but likely a close temporal association was present)	Levetiracetam/no improvement
					Veinoglobulins/only slight improvement
					Methylprednisolone/rapid, substantial improvement

31 [25]	Chan et al., 2021	44/M	-/nonsevere	Spontaneous, action-induced, posture-induced, and tactile stimuli sensitive myoclonus in the face, upper extremities, and lower extremities, wide-based, ataxic gait (12 d.a.o)	Methylprednisolone/partial improvement of spontaneous myoclonus; action-induced myoclonus persisted
					Clonazepam/partial improvement
					Levetiracetam/major improvement, discharged

32 [26]	Chaumont et al., 2020	62/M	HTN, DM/severe: Rq. ICU & MV	Postural and action myoclonus in upper limbs, ataxia (∼19 d.a.o)	Intravenous immunoglobulin/partial improvement of other non-MD-associated psychiatric and cognitive symptoms; myoclonus and ataxia persisted 3 weeks after discharge (final outcome NR)

33 [26]	Chaumont et al., 2020	72/M	HTN, DM, obesity, urothelial carcinoma in remission/severe: Rq. ICU & MV	Postural and action myoclonus in upper limbs, ataxia (∼34 d.a.o)	Intravenous immunoglobulin/partial improvement of other non-MD-associated psychiatric and cognitive symptoms; myoclonus and ataxia persisted 3 weeks after discharge (final outcome NR)

34 [26]	Chaumont et al., 2020	50/M	DM/severe: Rq. ICU & MV	Postural and action myoclonus in upper limbs, ataxia (∼48 d.a.o)	Intravenous immunoglobulin/partial improvement of other non-MD-associated psychiatric and cognitive symptoms; myoclonus and ataxia persisted 3 weeks after discharge (final outcome NR)

35 [26]	Chaumont et al., 2020	66/M	Obstructive sleep apnea/severe: Rq. ICU & MV	Postural and action myoclonus in upper limbs, ataxia (∼40 d.a.o)	Intravenous immunoglobulin/partial improvement of other non-MD-associated psychiatric and cognitive symptoms; myoclonus and ataxia persisted 3 weeks after discharge (final outcome NR)

36 [27]	Delorme et al., 2020	72/M	NR/nonsevere	Upper limbs myoclonus, and cerebellar ataxia (15 d.a.o)	Intravenous immunoglobulin/resolved

37 [28]	Dijkstra et al., 2020	44/M	Unremarkable/mild	Action‐induced myoclonic jerks in the face, arms, and trunk, intensified by tactile and acoustic stimuli, stuttering speech, prominent gait ataxia (∼14 d.a.o)	Methylprednisolone, intravenous immunoglobulin/gradually resolved within 2 months

38 [29]	Grimaldi et al., 2020	72/M	One episode of transient global amnesia (10 years ago)/nonsevere	Upper and lower limbs and trunk action tremor, ataxia, action- and stimuli-induced diffuse (but worse in proximal limbs) myoclonus (17 d.a.o)	Intravenous immunoglobulin/no significant improvement
					Intravenous methylprednisolone/considerable improvement

39 [30]	Sanguinetti et al., 2021	57/M	HTN, T2DM, DLP/nonsevere	Action-induced myoclonus in upper and lower extremities, opsoclonus, gait ataxia (at least 5 d.a.o)	Clonazepam, methylprednisolone/improved

40 [31]	Schellekens et al., 2020	48/M	Asymptomatic HIV+ with normal CD4+ T-cell count/nonsevere	Generalized myoclonus in trunk and limbs, present at rest but worsening both posturally and with action, cerebellar ataxia of arms and legs, and an ataxic gait (13 d.a.o)	Levetiracetam/improved

41 [32]	Borroni et al., 2020	54/F	HTN/nonsevere	Posture-induced myoclonus in diaphragm and left extremities (∼14 d.a.o)	Clonazepam/significant improvement

42 [32]	Borroni et al., 2020	80/M	-/nonsevere	Diaphragmatic myoclonus (23 d.a.o)	Levetiracetam/resolved
43 [33]	Khoo et al., 2020	65/F	Alzheimer's disease, osteoarthritis, gastroesophageal reflux disease/nonsevere	Upper and lower limbs, face, and tongue myoclonus, induced by tactile, visual, and auditory stimuli (7 d.a.o)	Levetiracetam, clonazepam/partial improvement
					Intravenous methylprednisolone/progressive improvement—discharged after 10 days

44 [34]	Muccioli et al., 2020	58/M	HTN/severe: Rq. ICU & MV	Multifocal myoclonus induced by action and tactile stimuli (at least 23 d.a.o)	Clonazepam, levetiracetam/significant improvement within 5 days
45 [35]	Ashraf et al., 2020	26/F	Obesity, asthma, PTSD, depression/asymptomatic	Right limb ataxia, ataxic gait (N/A)	Aspirin, clopidogrel, statin (for ischemic stroke)/NR
46 [36]	Balestrino et al., 2020	73/M	HTN, T2DM/nonsevere	Ataxic gait (at onset)	No additional treatment other than antiviral agents/resolved within 6 weeks

47 [27]	Delorme et al., 2020	60/F	Temporal lobe epilepsy (hippocampal sclerosis)/nonsevere	Limbs and gait ataxia (at onset)	Corticosteroid pulse therapy/resolved

48 [37]	Diezma-Martín et al., 2020	70/M	COPD/mild	Ataxia/tremor of voice, extremities, and gait, orthostatic tremor (17 d.a.o)	Clonazepam/slightly improved- discharged and continuously improved

49 [38]	Fadakar et al., 2020	47/M	-/nonsevere	Ataxia of extremities, gait, and trunk (3 d.a.o)	No additional treatment other than antiviral agents/significant improvement within two weeks

50 [39]	Fernández-Domínguez et al., 2020	74/F	HTN, follicular lymphoma/nonsevere	Gait ataxia (12–15 d.a.o)	Intravenous immunoglobulin/improvement

51 [40]	Gutiérrez-Ortiz et al., 2020	50/M	Asthma/nonsevere	Gait ataxia (3 d.a.o)	Intravenous immunoglobulin/resolved within two weeks

52 [41]	Hayashi et al., 2020	75/M	Alzheimer's disease/nonsevere	Ataxia of limbs and gait (at onset)	-/resolved after 2 days
53 [42]	Kopscik et al., 2020	31/M	-/nonsevere	Ataxia of limbs and gait (at onset)	Intravenous immunoglobulin/improvement

54 [43]	Lahiri et al., 2020	72/M	NR/NR	Ataxia (at onset)	NR/NR
55 [44]	Lantos et al., 2020	36/M	Remote strabismus/nonsevere	Gait ataxia (4 d.a.o)	Intravenous immunoglobulin/improvement

56 [45]	Lowery et al., 2020	45/M	HTN, DLP, Crohn's disease/severe: Rq. ICU & MV	Gait ataxia (14 d.a.o)	Intravenous immunoglobulin/delayed improvement
57 [46]	Manganotti et al., 2021	49/F	NR/nonsevere	Ataxia of limbs (14 d.a.o)	Intravenous immunoglobulin/improvement
58 [47]	Manganotti et al., 2021	50/F	-/nonsevere	Ataxia of gait and left upper limb (16 d.a.o)	Intravenous immunoglobulin/resolved

59 [48]	Perrin et al., 2021	64/M	HTN, DM, DLP, ESRD, sleep apnea, smoker/nonsevere	Ataxia (13 d.a.o)	Dexamethasone/improvement (followed by relapse)
					Intravenous immunoglobulin/rapid improvement

60 [48]	Perrin et al., 2021	53/F	-/severe: Rq. ICU & MV	Ataxia (NR, after extubation)	-/Spontaneous and gradual improvements 7 days later
61 [48]	Perrin et al., 2021	51/M	-/severe: Rq. ICU & MV	Ataxia (NR, after extubation)	-/Spontaneous and gradual improvements

62 [48]	Perrin et al., 2021	67/M	Kidney transplantation recipient (C3 glomerulopathy)/nonsevere	Ataxia (11 d.a.o)	Methylprednisolone/rapid improvement
63 [49]	Povlow et al., 2021	30/M	-/nonsevere	Ataxia of gait and extremities (at onset)	-/partial improvement within 10 days of hospitalization
64 [50]	Sartoretti et al., 2020	60/M	HTN, asthma/nonsevere	Ataxia (17 d.a.o)	Aspirin, atorvastatin (for ischemic stroke)/NR

## Data Availability

All relevant data are provided in the manuscript and the supplemental files; there are no additional data to be presented.

## Abbreviations

CKD: Chronic kidney disease
COPD: Chronic obstructive pulmonary disease
d.a.o: Days after onset
d.a.r: Days after recovery
DLP: Dyslipidemia
DM: Diabetes mellitus
ESRD: End-stage renal disease
F: Female
HLP: Hyperlipidemia
HTN: Hypertension
Hx: History
ICU: Intensive care unit (admission)
M: Male
MD: Movement disorder
MV: Mechanical ventilation
N/A: Not available
PLEX: Plasma exchange
POL: Predominant on the left
POR: Predominant on the right
PTSD: Posttraumatic stress disorder
Rq: Required
SARA: The scale for the assessment and rating of ataxia
T2DM: Type II diabetes mellitus
w/: With.

## Supplementary Materials

Supplementary Materials A direct link to video files of the three patients reported are included as supplementary data of this manuscript.
