# Supplementary material for: Movement Disorders Associated with COVID-19
Source: Parkinsons Dis. 2021 Nov 8;2021:3227753. doi: 10.1155/2021/3227753 (PMC8592762; doi:10.1155/2021/3227753)
Supplement: Supplementary Materials — A direct link to video files of the three patients reported are included as supplementary data of this manuscript. [file 3227753.f1.docx]

**Movement Disorders Associated with COVID-19**

Mehri Salari^a^, Bahareh Zaker Harofteh^a^, Masoud Etemadifar^b^, Nahad Sedaghat^b,c^, Hosein Nouri^b,c^,*

1. Department of Neurology, Shahid Beheshti University of Medical Sciences, Tehran, Iran
2. Alzahra Research Institute, Alzahra University Hospital, Isfahan University of Medical Sciences, Isfahan, Iran
3. Network of Immunity in Infection, Malignancy, and Autoimmunity (NIIMA), Universal Scientific Education and Research Network (USERN), Isfahan, Iran.

**Running title:**

COVID-19 and Movement Disorders

**Address for Correspondence:**

Hosein Nouri

Isfahan University of Medical Sciences, Isfahan, Iran.

Email: [Hosein.nouri.2018@gmail.com](mailto:Hosein.nouri.2018@gmail.com)

ORCID: 0000-0003-1808-0443

Tel: +989376709699

**Supplementary Material**

Provided below is the Google Drive link to a zip file containing three anonymized videos of the three patients reported in this article.

Please note that the video clips correspond numerically to the order in which the cases are reported, i.e., video 1 shows movement abnormalities in case no. 1, video 2 those in case no. 2, and video 3 those in case no. 3, as reported in the manuscript.

<https://drive.google.com/file/d/1QZWLJ_VjTgu8lMjbjPDgba0OgyMIVcVl/view?usp=sharing>
